# Quad-Band Plasmonic Perfect Absorber for Visible Light with a Patchwork of Silicon Nanorod Resonators

**DOI:** 10.3390/ma11101954

**Published:** 2018-10-12

**Authors:** Can Cao, Yongzhi Cheng

**Affiliations:** 1 School of Physics and Electronics, Central South University, Changsha 410083, China; 2 School of Information Science and Engineering, Wuhan University of Science and Technology, Wuhan 430081, China

**Keywords:** metamaterials (MMs), plasmonic perfect absorber, silicon resonator, visible light

## Abstract

In this paper, a plasmonic perfect absorber (PPA) based on a silicon nanorod resonator (SNRR) for visible light is proposed and investigated numerically. The proposed PPA is only a two-layer nanostructure consisting of a SNRR periodic array and metal substrate. The perfect absorption mainly originates from excitation of the localized surface plasmon resonance (LSPR) mode in the SNRR structure. The absorption properties of this design can be adjusted by varying the radius (*r*) and height (*h*) of the SNRR structure. What is more, the stronger quad-band absorption can be achieved by combing four different radius of the SNRR in one period as a super unit-cell. Numerical simulation indicates that the designed quad-band PPA can achieve the absorbance of 99.99%, 99.8%, 99.8%, and 92.2% at 433.5 THz, 456 THz, 482 THz, and 504.5 THz, respectively. Further simulations show that the proposed PPA is polarization-insensitive for both transverse electric (TE) and transverse magnetic (TM) modes. The proposed PPA can be a desirable candidate for some potential applications in detecting, sensing, and visible spectroscopy.

## 1. Introduction

Plasmonic perfect absorbers (PPAs) as an important branch of optical devices, which can absorb light completely at optical regime, have attracted increasing attention due to their promising applications in detecting, sensing, and energy harvesting [1,2,3,4,5,6,7,8]. The extraordinary optical absorption of the PPAs is usually attributed to intrinsic losses induced by the localized surface plasmon resonance (LSPR) in metamaterial (MM) [1,3,4,5,6,7,8]. The excitation of the LSPR stems from collective electron oscillations when the light interacts with patterned nanostructures or metallic nanoparticles [3,4,5,6]. The intrinsic losses can be enhanced significantly and achieve perfect absorption due to the LSPR excitation by optimizing the sizes, shapes, and arrangement configuration of nanostructures. Up to now, various PPA nanostructures have been demonstrated theoretically and experimentally in optical regimes due to their potential application [8,9,10,11,12,13,14,15,16,17,18]. Different resonance modes can be excited for the incident light and consequently producing critical EM losses in these PPAs nanostructures and finally cause perfect absorption. According to the different application requirements, these nanostructures are usually classified as broadband and narrow-/multiband PPAs in terms of the strong absorption bandwidth [8,9,10,11,12,13,14,15,16,17,18,19,20,21,22,23]. Generally, the broadband PPAs can be applicable in thermophotovoltaics and energy harvesting [8,9,10,11,12,13,14,15,16,17], while the narrow-/multiband ones are used in sensing and photodetection [18,19,20,21,22,23]. The most of these PPAs are based on bilayered or multilayered metal–dielectric (MD) nanostructures [24,25,26,27,28], which usually leads to higher cost and complexity in the fabrication process when large area production is required. This is because the PPA nanostructures are usually made of a noble metal (such as Ag, Au) that requires a complex fabrication process and long production cycles. Furthermore, such noble metals are difficult to integrate into current optoelectronic systems, which is still a challenge in practical applications. 

In recent years, the silicon nanostructure has drawn increasing attention due to its ability to support plasmon resonance in the optical regime [29,30,31,32,33,34]. Silicon is one of feasible high refractive index materials that has been widely used to design optical devices, such as solar cells [29], detectors [30], color filters [31,32], photonic waveguides [33], and absorbers [34]. It has been demonstrated that this plasmon resonance in the silicon nanostructure could be tuned to enhance light absorption in the infrared and visible ranges [34,35,36,37]. The silicon-based PPA is easy to integrate into mature compatible complementary metal-oxide-semiconductor (CMOS) technology [34]. What is more, the scattered light from the silicon nanostructure can be promoted to generate LSPR by exciting the free electrons of the plasmonic gold material. For example, Kazim et al. proposed a silicon based PPA for ultra-broadband absorption in the mid-infrared region, which can be fabricated using standard optical lithography [34]. Then, Liu proposed a PPA based on cavity mode using silicon patches array, multiband perfect absorption could be obtained in the infrared region [35]. More recently, PPAs based on a single silicon nanostructure were proposed and investigated and were shown to achieve a single-band perfect absorption in the visible region [36,37]. However, these PPAs still have drawbacks for polarization-sensitive or single-bands, which will limit their potential applications.

In this work, a plasmonic perfect absorber (PPA) based on a silicon nanorod resonator (SNRR) is proposed and investigated numerically in the visible spectrum. Firstly, we studied the perfect absorption property of the single-band PPA based on a single SNRR structure. The absorbance of the single-band PPA is greater than 99% at 441.5 THz due to the excitation of the LSPR mode under normal incidence. The perfect absorption properties can be adjusted dynamically by varying the geometric parameter of the nanostructure. Then, the quad-band perfect absorption (>90%) can be achieved by a patchwork of four SNRRs with different sizes. In comparison, this silicon nanostructure can be obtained using current nanofabrication technology. This quad-band PPA has great potential application in sensors, integrated photodetectors, and so on.

## 2. Structure Design and Simulation

**Figure 1:**
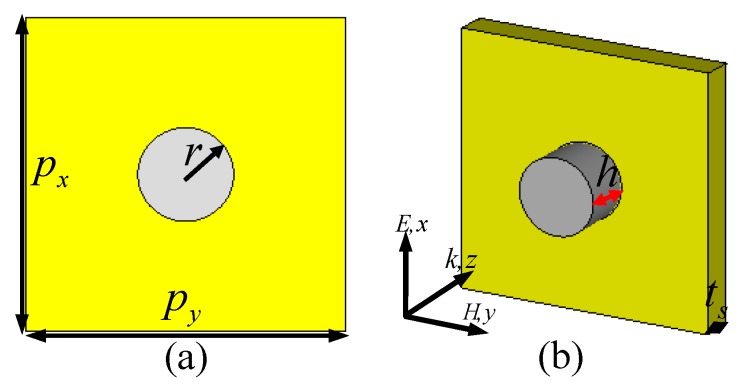
Schematic of the single-band plasmonic perfect absorber (PPA): (**a**) the front and (**b**) perspective view of the unit-cell nanostructure.

The unit-cell nanostructure of the single-band PPA is depicted in Figure 1a,b. Unlike traditional MDM nanostructures, the proposed PPA is only composed of two functional layers: the periodic array of the SNRR structure and a continuous gold film. The SNRR array was chosen as the dielectric resonator in the visible regime, which can support strong plasmonic resonances with different excitation modes [34,35,36,37]. The dielectric constant of the silicon is obtained from the experimental data [38]. A continuous gold film with thickness of 100 nm was selected as the metal substrate, which is described by a frequency-dependent Drude model from experimental data [39,40]. The 100 nm thickness of the gold substrate is much larger than the skin depth in the visible regime, thus the incident light can be canceled. As shown in Figure 1a,b, the period of the proposed PPA nanostructure is 400 nm; diffraction is avoided for frequencies up to 750 THz.

To study the performance of the proposed PPA, the full wave simulation was carried out based on the finite integration technique (FIT) of the CST Microwave Studio. In the simulation, the period boundary conditions were applied both in the *x*- and *y*-axis directions of the SNRR structure, and the open boundary condition was used along the *z*-axis direction. We can obtain the reflection coefficient *S*_11_(ω) of the proposed PPA by the CST simulation, thus, the absorbance can be expressed as *A*(ω) = 1 − *R*(ω) = |*S*_11_(ω)|^2^.

## 3. Results and Discussions

### 3.1. Narrowband PPA with Dependence of Silicon Nanostructure Geometric Dimension

**Figure 2:**
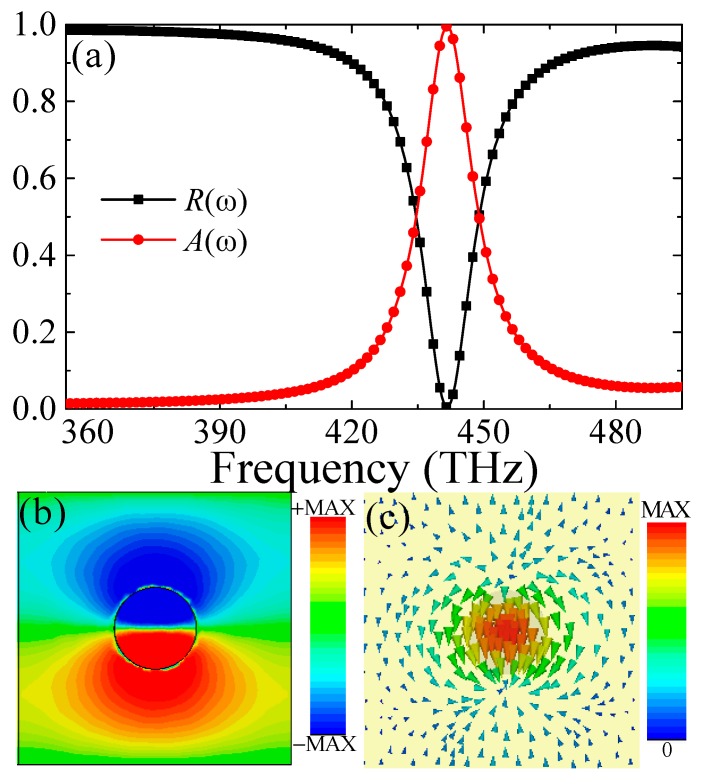
(**a**) The simulated reflectance (*R*(*ω*)) and absorbance (*A*(*ω*)) spectra of the single-band PPA, (**b,c**) the distributions of the electric field (*E_z_* in *x-y* plane) and surface current at 441.5 THz.

Firstly, we study the narrowband perfect absorption of the single SNRR structure with height (*h*) and radius (*r*) of 40 nm and 60 nm, respectively; and the corresponding reflectance and absorbance spectra are displayed in Figure 2a. It is clear that the reflectance of the PPA is near zero, and the corresponding absorption is up to 99.9% at 441.5 THz. Besides, the bandwidth of the full width at half maximum (FWHM) of the proposed PPA is only 14.5 THz, and the corresponding value of the Q-factor (Q = *f*/FWHM, where *f* is the resonance absorption frequency) is approximately 30.4. Thus, it is expected that the designed single-band PPA can serve as a detector and sensor due to its steep resonance with perfect absorption. It should be noticed that the designed single-band PPA is polarization-independent for both TE and TM waves under normal incidence due to its high symmetry of the unit-cell (not shown).

To obtain physical insight, we studied the distribution characteristics of the electric field and surface current at resonance, as shown in Figure 2b,c. One can see that the electric fields (*E_z_*) are mainly concentrated on the upper and lower areas of the SNRR structure at resonance, which is along the direction of incident electric field, as shown in Figure 2b. The enhanced electric field (*E_z_*) in the SNRR reveals that the larger opposite charges are accumulated on the edges of SNRR nanostructure. The surface charges in the SNRR structure oscillate like the electric dipole resonance response [41,42], which can be further validated by the surface current distribution, as shown in Figure 2c. Essentially, the electric dipole resonance is due to LSPR caused by the opposite charges accumulated on the upper and lower edges of the SNRR structure [1,2,3,7,8,19]. In this case, the electromagnetic (EM) field of the incident light also penetrates through the SNRR layers and is absorbed by the nanostructure [34]. The field profile caused by the LSPR mode excitation occurs in the nanostructure; the intrinsic loss usually takes place in the silicon and gold materials. Thus, this loss caused by the LSPR mode excitation should be mainly from the SNRR array due to the loss dielectric nature of silicon and gold material in the visible region. Consequently, incident light energy can be efficiently confined in the intermediate between the silicon nanostructure array and gold substrate layer and, therefore, no light is reflected back. That is, the introduced silicon nanostructure in the PLA significantly influences the coupling and confinement of the optical field. Following above analysis, we can conclude that the incident light energy mainly takes place in the SNRR structure and the continuous gold film. That is, the introduced silicon nanostructure in the PPA significantly influences the confinement of the EM field of the incident light.

**Figure 3:**
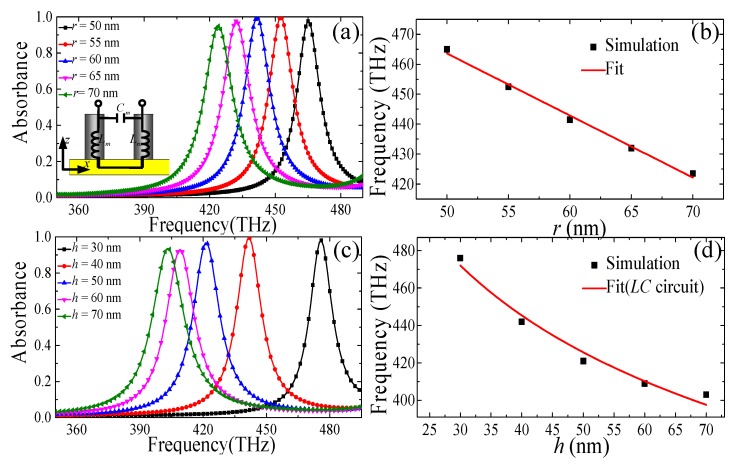
(**a**,**c**) Simulated absorbance of the PPA in different *r* and *h* of SNRR structure and (**b**,**d**) the dependences of the resonant absorption frequency are as functions of *r* and *h*.

To better understand the impact of the silicon nanostructure geometrical parameters on the absorption properties for the designed single-band PPA, a parametric study was conducted by numerical simulation. We studied the absorption properties by changing the radius (*r*) and height (*h*) of the SNRR structure, and the results are displayed in Figure 3a,b. As shown in Figure 3a, *r* is set from 50 to 55, 60, 65, and 70 nm while *h* was maintained at 40 nm. It can be observed that there is remarkable red-shift when increasing *r* of the SNRR structure, as shown in Figure 3a,b. In addition, the absorption peak firstly increases and then decreases slightly when *r* increases. A similar case also occurred when increasing *h* from 30 to 70 nm and fixing *r* to 40 nm, the frequency of the perfect absorption is shifted gradually to the lower frequency region as shown in Figure 3c,d. In addition, the absorption efficiency first increases and then decreases slightly. However, the absorbance is always over 92% when the changes of the *r* and *h* are in a certain range. For the operation frequency related to the geometric parameter of the single-band PPA, we can give a further interpretation by an equivalent *LC* circuit theory. Similar to the previous metal nanostructure [19,43,44], the resonance absorption frequency of our proposed PPA nanostructure is expressed as $f_{0}=\frac{1}{2\pi \cdot \sqrt{L_{m}C_{m}}}$. As shown in the inset of Figure 3a, the equivalent inductance *L_m_* is the magnetic inductance of two parallel rods based on the coil inductance which is approximately expressed as $L_{m}\approx \frac{A\mu_{0}h}{2\pi }\ln\frac{2h}{r}$, where *μ*_0_ is the permeability of free space, *r* and *h* are the radius and height of SNRR, respectively, and *A* is the numerical factor. The equivalent capacitance *C_m_* is the gap capacitance between the rods of two SNRRs, which is approximately expressed as $C_{m}\approx \frac{B\varepsilon_{0}\pi r}{p}$, where *ε*_0_ is the permittivity of free space, *p* is the periodicity of the PPA, and *B* is the numerical factor. According to the equivalent *LC* circuit theory, the resonance absorption frequency is approximately inversely proportional to the radius (*r*) and height (*h*) of the SNRR structure, which is confirmed by the above simulations (see Figure 3b,d). These results indicate that the geometrical parameters of the SNRR directly determine the operation frequency significantly and the absorption level slightly of the single-band PPA when the other parameters are fixed.

### 3.2. Quad-Band PPA

**Figure 4:**
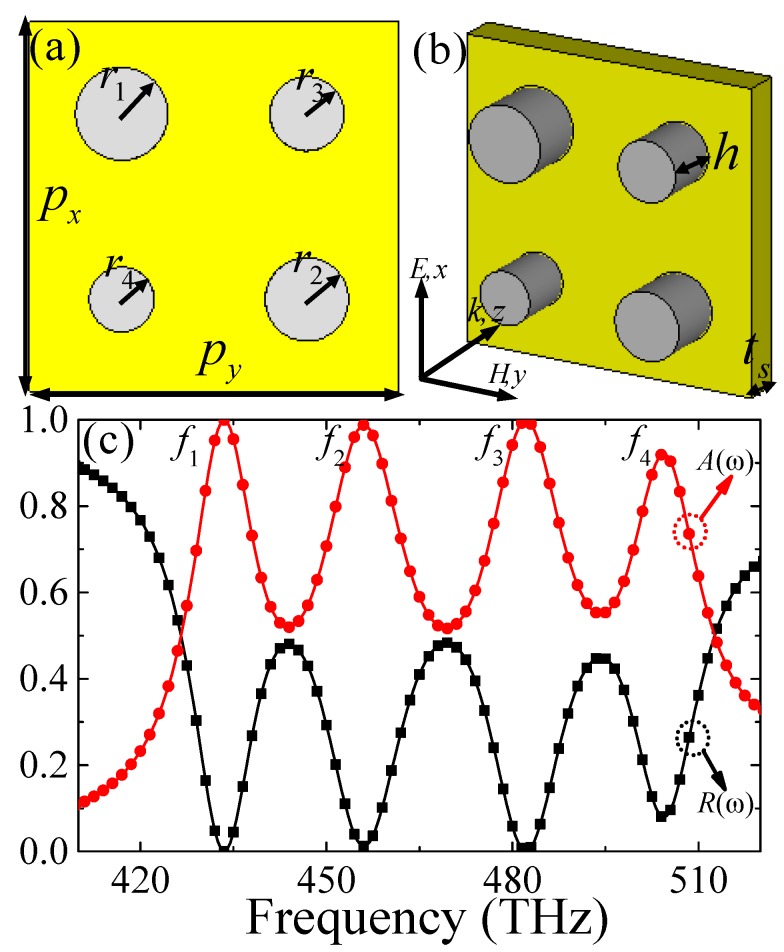
(**a**,**b**) The unit-cell nanostructure of quad-band PPA and (**c**) the corresponding simulated reflectance (*R*(*ω*)) and absorbance (*A*(*ω*)) spectra.

From the above simulation and analysis, it can be concluded that the perfect absorption can be adjusted easily by varying the radius (*r*) and height (*h*) of the SNRR structure. Thus, it inspires us to design a multiband PPA for visible light by patterning different SNRR with different *r* or *h* on a coplanar at several neighboring frequencies. In this section, to confirm the assumption, quad-band PPA based on a patchwork of four SNRR with different *r* as an example were studied numerically. Here, we present the schematic of the unit-cell nanostructure of the designed PPA, which is comprised of four SNRR with different *r* adhered to a continuous gold film, as shown in Figure 4a,b. To achieve quad-band perfect absorption, the optimized geometric parameters of the unit-cell nanostructure are given as *p_x_* = *p_y_* = 400 nm, *h* = 80 nm, *r*_1_ = 50 nm, *r*_2_ = 45 nm, *r*_3_ = 40 nm, *r*_4_ = 35 nm, and *t_s_* = 100 nm.

Figure 4c shows the reflectance and absorbance spectrum of the PPA based on the composite nanostructure under normal incident light, the four different resonance points are *f*_1_ = 433.5 THz, *f*_2_ = 456 THz, *f*_3_ = 482 THz, and *f*_4_ = 504.5 THz, respectively. At these resonance points, the reflectance is decreased to 0.01%, 0.2%, 0.2%, and 7.8%, and the corresponding absorbance is up to 99.99%, 99.8%, 99.8%, and 92.2%, respectively. In addition, the absorbance of the proposed PPA is greater than 50% from 426.5 THz to 512.5 THz, and the bandwidth of the FWHM is up to 86 THz, revealing a broadband stronger absorption property.

**Figure 5:**
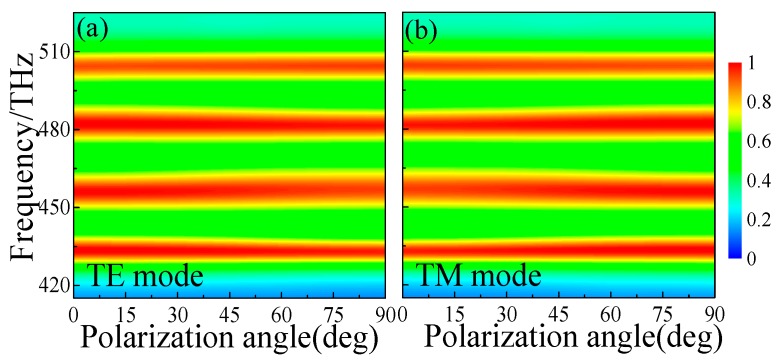
Simulated absorbance under normal incidence with different polarization angles at for (**a**) transverse electric (TE) and (**b**) transverse magnetic (TM) modes.

For practical application of the quad-band PPA, the absorption performance should be robust for different linear polarization angles for both TE and TM modes. Thus, we characterized the linear polarization angle dependences of the proposed quad-band PPA under normal incidence for both TE and TM modes. As shown in Figure 5a,b, it can be seen clearly that the high absorption level remains nearly unchanged when changing the polarization angle from 0° to 90° for both TE and TM modes. This means that the designed quad-band PPA is polarization-independent for normal incident light in the visible region.

**Figure 6:**
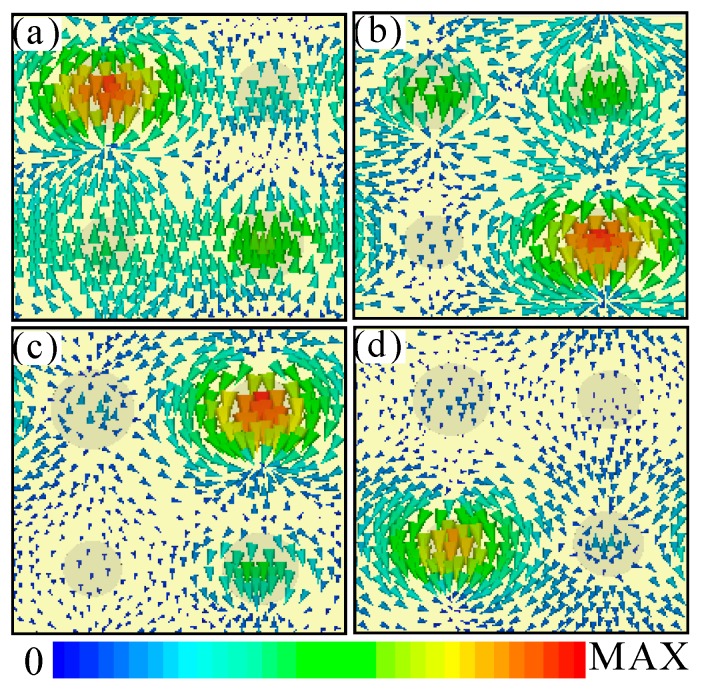
The distributions of surface current in the unit-cell nanostructure of the quad-band PPA at different resonance frequencies: (**a**) *f*_1_ = 433.5 THz, (**b**) *f*_2_ = 456 THz, (**c**) *f*_3_ = 482 THz, and (**d**) *f*_4_ = 504.5 THz.

Taking a further step, to illustrate the physical origin of the quad-band PPA, the distributions of surface current in the unit-cell were also simulated, as shown in Figure 6. It is well-known that the different resonance frequency is usually corresponding to the different size of the same resonator structure. Generally, the resonance absorption frequency (*f_m_*) is inversely proportional to size of the nanostructure. For our designed quad-band PPA, the absorption peak frequency (*f_m_*) is mainly determined by radius *r_i_* (*i* = 1, 2, 3, 4), and *f_m_* is inversely proportional to the *r_i_* of each SNRR structure (see Figure 3a). Accordingly, as shown in Figure 6a–d, the surface current distributions of the four resonances peaks are mainly concentered on the SNRR structure with four different sizes of *r*. It is clear that the distributions of surface current in the SNRR structure with smaller *r_i_* to higher frequency, while the larger *r*_i_ is the lower the frequency, which is consistent with the previous prediction. It also can be seen that the most of surface current of the quad-band PPA is distributed on the middle area of the SNRR structure, which is nearly equal to the single-band one. Obviously, the quad-band perfect absorption of the composite PPA is also mainly attributed to the intrinsic loss of silicon and gold material caused by excitation of the LSPR mode on the SNRR structure with different sizes of *r*. Therefore, the multiband and broadband strong absorption could be realized by appropriate design of the multi-SNRR structure with slightly different *r*.

## 4. Conclusions

In conclusion, we have proposed and demonstrated numerically a simple, visible PPA with a silicon nanostructure that was configured by a periodic array of the SNRR structure and a continuous gold film. The proposed PPA nanostructure exhibits a perfect absorption with an absorbance of 99.9% at 441.5 THz. The intrinsic loss of the silicon and gold material caused by the excitation of the LSPR mode contributed to the perfect absorption at resonance. The effects of different geometrical parameters on the absorption were compared in detail to study the characteristics of the proposed PPA. Furthermore, quad-band perfect absorption was achieved using four different-sized SNRR in a structural unit-cell. The absorbance of the proposed quad-band PPA was up to 99.99%, 99.8%, 99.8%, and 92.2% at 433.5 THz, 456 THz, 482 THz, and 504.5 THz, respectively. Moreover, the quad-band perfect absorption was observed to be polarization-independent for both TE and TM modes. This PPA based on silicon nanostructure can be fabricated easily by electron beam lithography (EBL) and reactive ion etching (RIE) [31,45]. So, a PPA with a simple design and better performance paves the way for the realization of all-silicon-based sensors, imagers, and spectroscopy applications, and provides chip-scale integration compatible with CMOS technology.
